# Health-related quality of life in patients with autoimmune hepatitis: A questionnaire survey

**DOI:** 10.1371/journal.pone.0204772

**Published:** 2018-10-04

**Authors:** Atsushi Takahashi, Kei Moriya, Hiromasa Ohira, Teruko Arinaga-Hino, Mikio Zeniya, Takuji Torimura, Masanori Abe, Akinobu Takaki, Jong-Hon Kang, Ayano Inui, Tomoo Fujisawa, Kaname Yoshizawa, Yoshiyuki Suzuki, Nobuhiro Nakamoto, Kazuhiko Koike, Hitoshi Yoshiji, Aya Goto, Atsushi Tanaka, Zobair M. Younossi, Hajime Takikawa, on behalf of the Japan AIH Study Group

**Affiliations:** 1 Department of Gastroenterology, Fukushima Medical University School of Medicine, Fukushima, Japan; 2 Department of Gastroenterology and Hepatology, Nara Medical University School of Medicine, Kashihara, Nara, Japan; 3 Department of Medicine, Kurume University School of Medicine, Kurume-shi, Fukuoka, Japan; 4 Sanno Medical Center, International University of Health and Welfare, Minato-ku, Tokyo, Japan; 5 Department of Gastroenterology and Metabology, Ehime University Graduate School of Medicine, Shitsukawa, Toon, Ehime, Japan; 6 Department of Gastroenterology and Hepatology, Okayama University Graduate School of Medicine, Dentistry, and Pharmaceutical Sciences, Kita-ku, Okayama City, Japan; 7 Center for Gastroenterology, Teine Keijinkai Hospital, Teine-ku, Sapporo, Japan; 8 Department of Pediatric Hepatology and Gastroenterology, Saiseikai Yokohama Tobu Hospital, Tsurumi-ku, Yokohama City, Kanagawa, Japan; 9 Department of Gastroenterology, National Hospital Organization, Shinshu Ueda Medical Center, Ueda City, Nagano, Japan; 10 Department of Hepatology, Toranomon Hospital, Minato-ku, Tokyo, Japan; 11 Department of Internal Medicine, Keio University School of Medicine, Shinjuku-ku, Tokyo, Japan; 12 Department of Gastroenterology and Hepatology, The Third Hospital of Jikei University School of Medicine, Komae-shi, Tokyo, Japan; 13 Center for Integrated Science and Humanities, Fukushima Medical University, Fukushima, Japan; 14 Department of Medicine, Teikyo University School of Medicine, Itabashi-ku, Tokyo, Japan; 15 Betty and Guy Beatty Center for Integrated Research, Inova Health System, Falls Church, VA, United States of America; Nihon University School of Medicine, JAPAN

## Abstract

**Aim:**

Health-related quality of life is impaired in patients with autoimmune hepatitis, but the association between health-related quality of life and patients’ backgrounds remains unknown. We assessed health-related quality of life in patients with autoimmune hepatitis and identified factors associated with its impairment.

**Methods:**

We assessed health-related quality of life in patients with autoimmune hepatitis, patients with chronic hepatitis C, and healthy subjects using the Japanese version of the Chronic Liver Disease Questionnaire and the 36-Item Short Form Survey. We compared health-related quality of life in patients with autoimmune hepatitis with that of patients with chronic hepatitis C and healthy subjects.

**Results:**

A total of 265 patients with autoimmune hepatitis, 88 patients with chronic hepatitis C, and 97 healthy subjects were enrolled; most patients were women. The median ages of patients were 65, 66, and 57 years, respectively. Of these patients with autoimmune hepatitis, 10.6% and 57.0% had cirrhosis and comorbid diseases, respectively. The overall Chronic Liver Disease Questionnaire scores (5.5 vs. 6.2, *P* < 0.001) and physical (48.1 vs. 54.2, *P* < 0.001) and mental (51.8 vs. 55.0, *P* = 0.004) component summaries of 36-Item Short Form Survey were significantly lower in patients with autoimmune hepatitis than in healthy subjects, and similar to scores in patients with chronic hepatitis C. Having cirrhosis, comorbid diseases, and treatment for autoimmune hepatitis were associated with impaired health-related quality of life among patients with autoimmune hepatitis. In particular, prednisolone use was associated with lower scores on the worry domain of the Chronic Liver Disease Questionnaire.

**Conclusions:**

Patients with autoimmune hepatitis showed impairment in health-related quality of life, which was associated with not only disease progression, but also comorbid diseases and treatment. Ways to improve health-related quality of life should be considered in patients with AIH when disease outcome is not favorable and when using prednisolone.

## Introduction

Autoimmune hepatitis (AIH) is usually well controlled by immunosuppressive therapy such as corticosteroids and azathioprine. Repeated relapse of AIH is associated with poor prognosis [1]. Physicians usually focus on patients’ treatment response or the side effects of immunosuppressive therapy but overlook or misunderstand the quality of life in patients with AIH, especially in well-controlled patients. In addition to objective evaluative indexes, such as mortality rate, patient-reported outcomes have become an important factor in the management of patients with various chronic diseases. A recent cohort study reported that higher health-related quality of life (HrQoL) was associated with survival in patients with chronic liver disease [2].

A previous study using the 36-Item Short Form Survey (SF-36) and the Multidimensional Fatigue Index-20 showed that HrQoL was more impaired in patients with AIH than in healthy controls [3]. In addition, a recent study using the 12-Item Short Form Survey (SF-12), the patient health questionnaire, and the Generalized Anxiety Disorder Screener reported that mental well being in patients with AIH was significantly reduced compared with the general population [4]. However, the associations between each component of the HrQoL and other patient factors, such as laboratory findings, comorbid diseases, and treatment remain unclear.

The Chronic Liver Disease Questionnaire (CLDQ) was first proposed as a disease-specific instrument to evaluate HrQoL [5] and has been widely used to assess HrQoL among patients with different chronic liver diseases [6–8]. The present study used the Japanese version of the CLDQ and SF-36 to measure HrQoL in patients with AIH and examined the association between patient background variables and HrQoL in patients with AIH.

## Materials and methods

### Study population

This study was conducted among all members of the Autoimmune Hepatitis Study Group, a subgroup of the Intractable Hepato-biliary Disease Study Group in Japan, and the Liver Disease Patient Association in Tokyo. The diagnosis of AIH was made based on Japanese diagnostic guidelines [9]. Chronic hepatitis C (CHC) and liver cirrhosis were determined using histological, biochemical, and/or imaging studies. Patients with AIH and patients with CHC were recruited consecutively at each hospital and clinic. Patients with the presence or history of malignant disease and any mental disorder were excluded. Among patients with CHC, 32 patients were HCV RNA negative by direct-acting antiviral agents (25 patients) and interferon (7 patients). Patients with CHC patients who had been treated with interferon within 6 months and patients with CHC with liver cirrhosis were also excluded. Healthy subjects were enrolled after agreement to join this study. Subjects were enrolled between August 2015 and September 2016, and written informed consent was obtained from all subjects. This study protocol conformed to the ethical guidelines of the 1975 Declaration of Helsinki and was approved by the Ethics Committee of Fukushima Medical University (no. 2130).

### Questionnaire

We asked participants to complete two self-reported questionnaires: the Japanese versions of the CLDQ and the SF-36. The original version of the CLDQ includes 29 items and is divided into six subdomains: abdominal symptoms (three items), fatigue (five items), systemic symptoms (five items), activity (three items), emotional function (eight items), and worry (five items) [5]. The SF-36 Japanese version consists of 36 items and is divided into eight multi-item scales on physical functioning, role physical (role limitations as a result of physical health), bodily pain, general health perception, vitality, social functioning, role emotion (role limitations as a result of mental problems), and mental health [10]. The original Japanese version of the SF-36 can be aggregated into two summary scores: the mental component summary score and the physical component summary score. The Japanese versions of the CLDQ and SF-36 were previously published and statistically validated prior to the present study [6, 10]. Moreover, three component models of the SF-36, that is, the physical, mental, and role/social component summary, have been validated in Japan [11]. In addition to the CLDQ and SF-36, we collected data on age, body mass index, laboratory findings, comorbid diseases (i.e., hypertension, diabetes, osteoporosis, dyslipidemia, primary biliary cholangitis, rheumatoid arthritis, endocrine disorder, collagen disease, cardiovascular disease and respiratory disease), and prednisolone treatment. The fibrosis-4 (FIB-4) index was determined using the following formula [12]: Fib-4 index = age (years) × aspartate aminotransferase (AST) [U/L] / (platelet count [10^9^/L] × (alanine aminotransferase [ALT] [U/L])^1/2^).

### Statistical analyses

Results are presented as medians (interquartile range). Patients with AIH and CHC were compared using the Mann-Whitney *U*-test for continuous variables. Correlations between variables were measured using Spearman’s rank correlation to examine the association between patient background and HrQoL in patients with AIH. The age and sex-adjusted odds ratios (ORs) of values less than the median scores of healthy subjects among patients with AIH were evaluated by logistic regression. Specifically, dependent variables included each CLDQ and SF-36 item, the independent variable of interest was patients with AIH versus healthy controls, and adjustment variables were age and sex. Moreover, another logistic regression analysis was repeated among patients with AIH to elucidate the effects of patient background factors. For this model, dependent variables were each CLDQ and SF-36 item, the independent variable was presence versus absence of each background factor (cirrhosis, comorbidities, and prednisolone treatment), and adjustment variables were age and sex. The same analysis was repeated three times for each background factor. *P* < 0.05 was considered statistically different. Statistical analysis was performed using SPSS 17.0 for Windows (SPSS, Inc., Chicago, IL, USA).

## Results

### Clinical and demographic data

A total of 265 patients with AIH, 88 patients with CHC, and 97 healthy subjects were enrolled. The median ages of patients were 65, 66, and 57 years, respectively. The mean age of patients with AIH was significantly higher than that of healthy subjects. The proportion of women was higher in among patients with AIH than in patients with CHC (87.5% vs 73.9%, *P* = 0.002). Levels of AST, ALT, and alkaline phosphatase (ALP) were significantly lower in patients with AIH than in patients with CHC. Of the 265 patients with AIH, 28 (10.6%) had cirrhosis, and 151 (57.0%) had a comorbid disease. In the 151 patients with a comorbid disease, hypertension was the most frequent disease (19.9%), followed by diabetes (17.2%), osteoporosis (15.9%), dyslipidemia (13.9%), and chronic thyroiditis (10.6%). Primary biliary cholangitis and rheumatoid arthritis were seen in 14 (9.3%) patients and 12 (7.9%) patients, respectively. Table 1 shows demographic data of all subjects.

**Table 1:** Characteristics of subjects.

	AIH (n = 265)	CHC (n = 88)	Healthy (n = 97)	*P* (vs. CHC)	*P* (vs. healthy)
Sex (women)	87.5% (232)	73.9% (65)	80.4% (78)	0.002	0.188
BMI (kg/m^2^)	22.5 (20.2–25.2)	22.4 (20.8–24.9)	22.1 (20.9–24.1)	0.917	0.290
Age (years)	65 (55–73)	66 (60–73)	57 (51–62)	0.090	< 0.001
AST (U/L)	23 (18–31)	27 (22–42)	-	< 0.001	-
ALT (U/L)	17 (12–27)	22 (17–42)	-	< 0.001	-
ALP (U/L)	213 (164–262)	269 (204–334)	-	< 0.001	-
Plt (×10^4^/μL)	18.7 (14.6–22.7)	16.9 (13.3–20)	-	0.036	-
Use of PSL	70.6% (187)	-	-	-	-
FIB-4 index	2.01 (1.43–2.88)	2.31 (1.54–3.29)	-	0.066	-
Cirrhosis	10.6% (28)	-	-	-	-
Comorbid disease (case)	57.0% (151)	54.5% (48)		0.690	
Hypertension (case)	11.3% (30)	23.9% (21)		0.006	
Diabetes (case)	9.8% (26)	9.1% (8)		0.992	
Osteoporosis (case)	9.1% (24)	4.5% (4)		0.259	

Data are median (interquartile range) for continuous variables, percentage values for categorical variables. AIH, autoimmune hepatitis; CHC, chronic hepatitis C; BMI, body mass index; AST, aspartate aminotransferase; ALT, alanine aminotransferase; ALP, alkaline phosphatase; Plt, platelet; PSL, prednisolone.

### CLDQ domain scores

The overall scores and all the domain scores for CLDQ were significantly lower in patients with AIH patients than in healthy subjects (Table 2). There were no significant differences between patients with AIH patients and patients with CHC. These results were similar among AIH patients without cirrhosis (S1 Table). Compared with healthy subjects, the age and sex-adjusted ORs for being less than the median scores of the healthy subjects were significantly higher for the overall score and the scores of the five subdomains, except for the abdominal domain, among patients with AIH (Table 3). Among the subdomains, the age and sex-adjusted ORs among patients with AIH were highest in the worry domain.

**Table 2:** CLDQ and SF-36 scores among patients with AIH, patients with CHC, and healthy subjects.

	AIH (n = 265)	CHC (n = 88)	Healthy (n = 97)	*P*-value (AIH vs. CHC)	*P*-value (AIH vs. Healthy)	*P*-value (CHC vs. Healthy)
CLDQ						
Overall	5.5 (4.8–6.0)	5.6 (5.0–5.8)	6.2 (5.7–6.5)	0.782	< 0.001	< 0.001
Abdominal	6.0 (5.3–6.7)	6.0 (5.6–6.7)	6.3 (5.7–7.0)	0.372	0.100	0.031
Fatigue	5.2 (4.4–5.8)	5.3 (4.6–5.8)	5.8 (5.4–6.2)	0.520	< 0.001	< 0.001
Systemic	5.6 (4.8–6.2)	5.6 (5.0–6.0)	6.2 (5.8–6.6)	0.783	< 0.001	< 0.001
Activity	5.7 (5.0–6.3)	6.0 (5.5–6.3)	6.3 (6.0–7.0)	0.296	< 0.001	< 0.001
Emotions	5.4 (4.4–6.0)	5.5 (4.8–5.9)	5.9 (5.3–6.3)	0.841	< 0.001	< 0.001
Worry	5.4 (4.4–6.0)	5.6 (4.6–6.0)	6.8 (6.3–7.0)	0.272	<0.001	< 0.001
SF-36						
Physical functioning	90 (70–95)	90 (75–95)	95(90–100)	0.468	< 0.001	< 0.001
Role physical	87.5(60.9–100)	100 (75–100)	100 (87.5–100)	0.063	< 0.001	0.004
Bodily pain	84 (62–100)	74 (61–100)	84 (72–100)	0.150	0.036	0.002
General health	52 (42.4–62)	53.5 (45–62)	72 (62–84.5)	0.305	< 0.001	< 0.001
Vitality	62.5 (43.8–75)	62.5 (50–68.8)	75 (62.5–81.3)	0.700	< 0.001	< 0.001
Social functioning	100 (62.5–100)	100 (75–100)	100 (87.5–100)	0.160	< 0.001	0.015
Role emotion	83.3 (66.7–100)	100 (83.3–100)	100 (91.7–100)	0.008	< 0.001	0.008
Mental health	70 (55–85)	75 (60–85)	80 (70–90)	0.399	< 0.001	0.005
PCS	48.1 (38.1–54.3)	47.1 (38.3–53.2)	54.2 (49.6–57.2)	0.490	< 0.001	< 0.001
MCS	51.8 (44.3–57.4)	51.3 (46.4–54.8)	55.0 (48.2–60.5)	0.310	0.004	< 0.001
RCS	49.6 (38.1–56.1)	53.9 (47.0–58.0)	51.8 (47.2–55.5)	0.003	0.049	0.166

Data are shown as median (interquartile range). CLDQ, Chronic Liver Disease Questionnaire; SF-36, 36-Item Short Form Survey; AIH, autoimmune hepatitis; CHC, chronic hepatitis C; PCS, physical component summary; MCS, mental component summary; RCS, role/social component summary.

**Table 3:** Age-sex adjusted odds ratios of being less than medians score of healthy subjects among AIH patients (n = 265).

	Odds ratio	95% confidence interval.	P-value
CLDQ			
Overall	4.81	2.86–8.08	<0.001
Abdominal	1.36	0.84–2.21	0.212
Fatigue	3.32	2.02–5.47	<0.001
Systemic	3.03	1.84–4.97	<0.001
Activity	4.17	2.47–7.03	<0.001
Emotions	1.95	1.20–3.18	0.008
Worry	11.80	6.32–22.01	<0.001
SF-36			
Physical functioning	2.19	1.31–3.66	0.003
Role physical	2.29	1.38–3.79	0.001
Bodily pain	1.42	0.86–2.33	0.171
General health	5.58	3.27–9.53	< 0.001
Vitality	2.82	1.72–4.63	< 0.001
Social functioning	2.34	1.39–3.95	0.001
Role emotion	3.47	2.04–5.90	< 0.001
Mental health	2.79	1.69–4.58	< 0.001
PCS	2.45	1.47–4.07	0.001
MCS	2.09	1.26–3.46	0.004
RCS	1.30	0.80–2.11	0.287

Logistic regression analysis was used (dependent variable: each CLDQ and SF-36 item, independent variable of interest: patients with AIH versus healthy subjects, adjustment variables: age and sex).

CLDQ, Chronic Liver Disease Questionnaire; SF-36, 36-Item Short Form Survey; PCS, physical component summary; MCS, mental component summary; RCS, role/social component summary.

Regarding the associations between clinical and demographic data and HrQoL among patients with AIH, age was negatively correlated with the scores of the systemic domain (*rs* = -0.16, *P* < 0.05) and the activity domain (*rs* = -0.18, *P* < 0.05). Platelet count was positively correlated with overall scores (*rs* = 0.12, *P* < 0.05), scores of the systemic domain (*r* = 0.17, *P* < 0.05), and scores of the activity domain (*rs* = 0.18, *P* < 0.05) (Table 4).

**Table 4:** Associations between backgrounds of patients with AIH and CLDQ or SF-36 scores.

	Coefficient of correlation [rs]					
	Age	Duration	AST	ALT	ALP	Platelet
CLDQ						
Overall	-0.02	0.02	-0.05	-0.08	-0.06	0.12*
Abdominal	0.10	0.05	0.05	0.03	-0.04	0.09
Fatigue	0.00	0.00	-0.02	-0.04	-0.07	0.08
Systemic	-0.16*	-0.06	-0.09	-0.08	-0.18*	0.17*
Activity	-0.18*	-0.06	-0.06	-0.04	-0.07	0.18*
Emotions	0.05	0.06	-0.01	-0.05	0.00	0.07
Worry	0.03	0.08	-0.07	-0.12	0.02	0.08
SF-36						
Physical functioning	- 0.45*	-0.13*	-0.11	-0.00	-0.12*	0.27*
Role physical	-0.24*	-0.04	-0.10*	-0.03	-0.10*	0.21*
Bodily pain	-0.06	-0.06	-0.06	0.05	-0.16*	0.12
General health	-0.02	-0.05	-0.07	-0.08	-0.01	0.12
Vitality	0.01	-0.02	0.02	-0.01	0.03	0.07
Social functioning	0.05	0.06	0.01	-0.01	0.02	0.09
Role emotion	-0.17*	-0.03	-0.12*	-0.06	-0.07	0.18*
Mental health	0.07	0.04	0.01	-0.01	-0.01	0.03
PCS	-0.45*	-0.18*	-0.14*	-0.01	-0.16*	0.24*
MCS	0.30*	0.07	0.08	-0.01	0.08	-0.06
RCS	-0.15*	-0.04	-0.16*	-0.10	-0.10	0.07

AST, aspartate aminotransferase; ALT, alanine aminotransferase; ALP, alkaline phosphatase; CLDQ, Chronic Liver Disease Questionnaire; SF-36, 36-Item Short Form Survey; PCS, physical component summary; MCS, mental component summary; RCS, role/social component summary.

*P < 0.05

**Fig 1:**
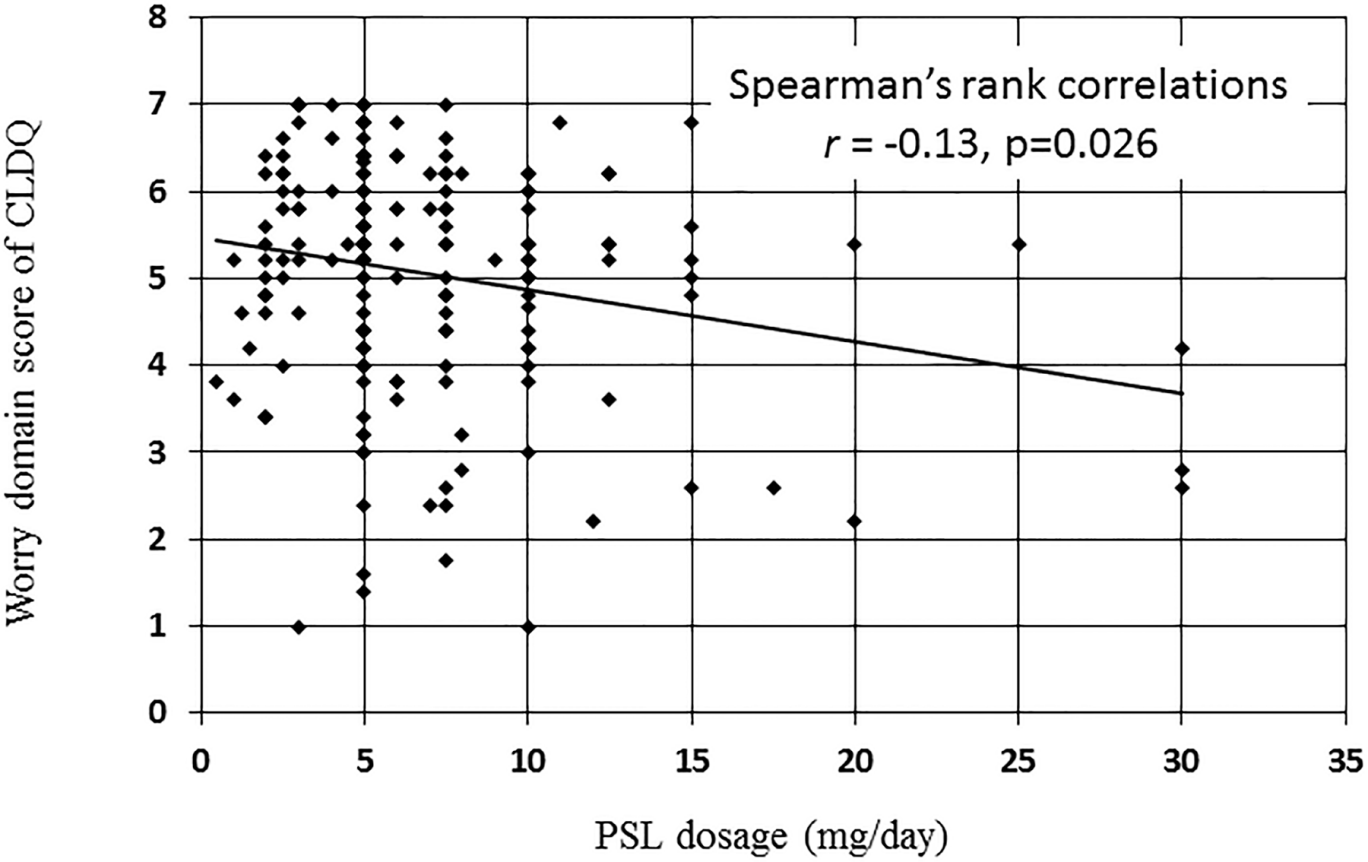
Association between prednisolone dosage and worry domain score of the Chronic Liver Disease Questionnaire.

For overall scores, the age and sex-adjusted ORs for being less than the median scores of patients with AIH were significantly higher among those with cirrhosis than those without cirrhosis (OR 2.87, *P* = 0.016) (Table 5). The adjusted ORs for overall scores were also significantly higher in patients with AIH with a comorbid disease than in those without a comorbid disease (OR 1.76, *P* = 0.036). Similar findings were seen for scores in the systemic domain (OR 1.93, *P* = 0.015). The adjusted OR of the worry domain was significantly higher in the patients with AIH treated with prednisolone (OR 1.79, *P* = 0.038) than in those who did not receive prednisolone. Moreover, the dosage of prednisolone was negatively correlated with the worry domain score (*rs* = -0.13, *P* = 0.026) (Fig 1).

**Table 5:** Among patients with AIH, age- and sex-adjusted odds ratios of being less than medians scores among patients with cirrhosis, a comorbid condition, and prednisolone treatment (total n = 265).

	Cirrhosis (n = 28)			Comorbid disease (n = 151)			Prednisolone treatment (n = 187)		
	Odds ratio	95% CI	p-value	Odds ratio	95% CI	p-value	Odds ratio	95% CI	p-value
CLDQ									
Overall	2.87	1.21–6.81	0.016	1.76	1.04–2.97	0.036	1.37	0.80–2.35	0.248
Abdominal	1.35	0.60–3.01	0.469	1.39	0.80–2.42	0.249	0.63	0.36–1.11	0.108
Fatigue	1.31	0.59–2.88	0.508	1.59	0.94–2.69	0.084	0.63	0.37–1.08	0.091
Systemic	1.88	0.84–4.21	0.125	1.93	1.14–3.27	0.015	0.86	0.51–1.47	0.580
Activity	2.22	0.96–5.17	0.063	1.60	0.95–2.71	0.080	0.89	0.52–1.53	0.677
Emotions	2.00	0.90–4.50	0.090	1.53	0.90–2.59	0.117	0.98	0.57–1.68	0.945
Worry	2.15	0.96–4.82	0.062	1.17	0.70–1.98	0.547	1.79	1.03–3.11	0.038
SF-36									
Physical functioning	3.02	1.19–7.65	0.020	1.52	0.86–2.68	0.147	1.68	0.90–3.13	0.104
Role physical	1.62	0.72–3.67	0.247	2.15	1.23–3.75	0.007	1.01	0.56–1.81	0.986
Bodily pain	1.25	0.56–2.78	0.592	1.84	1.07–3.14	0.027	1.03	0.58–1.83	0.926
General health	1.72	0.78–3.78	0.182	1.88	1.09–3.24	0.023	1.42	0.79–2.56	0.243
Vitality	1.22	0.54–2.77	0.628	1.07	0.63–1.82	0.800	1.71	0.94–3.11	0.078
Social functioning	0.57	0.25–1.30	0.181	1.19	0.71–2.02	0.509	1.07	0.60–1.90	0.823
Role emotion	1.42	0.64–3.15	0.392	2.13	1.21–3.75	0.008	0.94	0.52–1.69	0.823
Mental health	0.99	0.43–2.29	0.985	1.45	0.84–2.51	0.182	1.05	0.58–1.91	0.860
PCS	7.99	2.48–25.72	< 0.001	1.20	0.66–2.17	0.547	1.04	0.55–1.97	0.903
MCS	1.55	0.67–3.59	0.305	1.06	0.61–1.85	0.829	1.49	0.82–2.73	0.195
RCS	1.64	0.71–3.78	0.246	1.24	0.73–2.12	0.431	0.97	0.54–1.73	0.906

Logistic regression analysis was used (dependent variable: each CLDQ and SF36 item, independent variable of interest: presence versus absence of each background, adjustment variables: age and sex). CI, confidence interval; CLDQ, Chronic Liver Disease Questionnaire; SF-36, 36-Item Short Form Survey; PCS, physical component summary; MCS, mental component summary; RCS, role/social component summary.

### SF-36 scale and summary scores

The SF-36 scores of the AIH patients, CHC patients, and healthy subjects are shown in Table 2. Compared to the healthy subjects, all component and summary scores of SF-36 were significantly lower in the AIH patients. The scores of the role emotion summary and the role/social component summary in the AIH patients were significantly lower than those of the CHC patients (Table 2). These results were almost similar among AIH patients without cirrhosis (S1 Table).

Compared with the healthy subjects, the age and sex-adjusted ORs for being less than the median scores of the healthy subjects were significantly higher in the 7 components except bodily pain and the two-summary scores among the AIH patients (Table 3). Among the SF-36 scores, the adjusted ORs were highest in general health perception.

The correlation between SF-36 score and AIH patient background was evaluated as well as the correlation between CLDQ score and AIH patient background. Age was negatively correlated with the scores of three components (physical functioning, role physical, role emotion) and two summary scores (physical and role/social component). Similarly, disease duration was also negatively correlated with the score of physical functioning and two summary scores (physical and role/social component). Although there was no association between ALT and SF-36 scores, AST was negatively correlated with the scores of two components (role physical and role emotion) and two summary scores (physical and role/social component). Moreover, ALP was negatively correlated with the scores of physical functioning, role physical, bodily pain and physical component summary. The platelet count was positively correlated with the scores of three components (physical functioning, role physical, role emotion) and the physical component summary (Table 4).

The age and sex-adjusted ORs for being less than the median scores of the AIH patients were significantly higher in the AIH patients with cirrhosis rather than without cirrhosis for physical functioning (OR 3.02, *P* = 0.020) and the physical component summary (OR 7.99, *P* < 0.001) (Table 5). The adjusted ORs were also significantly higher in the AIH patients with comorbid disease rather than without comorbid disease for role physical (OR 2.15, *P* = 0.007), bodily pain (OR 1.84, *P* = 0.027), general health (OR 1.88, *P* = 0.023) and the role emotion (OR 2.13, *P* = 0.008). Prednisolone treatment was not associated with the scores of SF-36 among the patients with AIH.

## Discussion

The present study evaluated HrQoL of patients with AIH using a disease-specific (CLDQ) and a generic (SF-36) HrQoL instrument. Of note, this is the first assessment of HrQoL in patients with AIH using the CLDQ. We found that HrQoL was more impaired in patients with AIH than in healthy subjects and was relatively comparable to that of patients with CHC according to both the CLDQ and the SF-36. Additionally, we found that HrQoL in patients with AIH was closely associated with factors such as age, the presence of cirrhosis, comorbid diseases, and treatment with prednisolone. This study is also the first to elucidate the association between the various HrQoL factors and patient background factors in patients with AIH.

A recent study reported HrQoL impairment, as assessed with the Japanese version of the CLDQ, in Japanese patients with chronic viral hepatitis; the CLDQ scores of these patients were lower compared to those of the present study [7]. Moreover, there were significant differences in the scores for role emotion summary and the role/social component summary of the SF-36 in this study. HCV eradication improves HrQoL in patients with CHC [13]. One third or more of patients with CHC in the present study were HCV RNA negative; therefore, HrQoL of patients with CHC may be better than that of previously reported patients with chronic viral hepatitis [7].

Previous studies found HrQoL impairment in patients with chronic liver disease including AIH [2–3]. However, the causes of HrQoL impairment were not elucidated. Another study showed that depression and anxiety in patients with AIH were related to the progression of the liver disease [4]. The present study found that not only disease conditions such as laboratory findings or cirrhosis, but also comorbid diseases and treatment with prednisolone were associated with impaired physical condition as well as mental and emotional health in patients with AIH.

Patients’ mental state may be the HrQoL factor that clinicians focus on most, because a mental disorder is a well-known prednisone-related side effect [14]. Moreover, psychosocial factors are associated with treatment adherence and response [15]. Depression and severe anxiety have been found to be more frequent in patients with AIH compared to the general population [4]. In the present study, the worry domain had the highest OR in the CLDQ, and prednisolone treatment was significantly associated with the worry domain. Notably, a positive correlation between prednisolone dosage and the worry domain score was identified for the first time in the present study. Although the SF-36 also showed the mental component score to be impaired, there was no significant correlation between patient background and the mental component score. The SF-36 is a generic questionnaire for the evaluation of HrQoL; therefore, the SF-36 is not capable of assessing liver disease-specific aspects of HrQoL. The CLDQ was designed as a liver-disease specific instrument for the evaluation of HrQoL. Compared with the SF-36, the CLDQ discriminates HrQoL better between cholestatic liver disease patients with early disease and those with advanced disease [6]. Interestingly, it has also been reported that the effect of disease severity on emotional health is picked up relatively well by the emotional function domain of the CLDQ but not at all by the SF-36 [6]. These observations may imply that findings regarding mental factors differ according to the evaluation method used. Therefore, differences in evaluation methods should be considered when assessing HrQoL in patients with AIH.

In addition to cirrhosis and prednisolone treatment, comorbid diseases are an important background factor for HrQoL in patients with AIH. In the present study, complications were associated with physical factors in both the CLDQ and SF-36. Interestingly, ALP levels were negatively correlated with the systemic domain score of the CLDQ, and with physical functioning, role physical, bodily pain, and the physical component summary in the SF-36. These results may imply that comorbid diseases such as rheumatoid arthritis, bone fracture, or primary biliary cholangitis may influence the above factors of HrQoL in patient with AIH. On the other hand, previous studies reported HrQoL impairment in patients with diabetes [16], which is a major complication of prednisolone treatment. Therefore, treatment complications including diabetes should be controlled to avoid HrQoL impairment in patients with AIH. Although we could not evaluate the total dose of prednisolone during clinical course in the present study, these doses should be assessed to prevent complications, as long-term treatment with longer disease duration may be associated with complications.

The strengths of our study were the sample size of the patients with AIH, who were recruited from all over Japan, and evaluation of HrQoL by using the CLDQ and the SF-36. As a limitation of the current study, 187 (70.6%) AIH patients were being treated with prednisolone, and 166 (62.6%) were in remission and being treated by prednisolone at a dose of ≤10 mg/day. In this regard, one may consider that only patients with AIH at presentation before treatment should have been included to clarify the impact of the disease on HrQoL without a treatment effect. However, in the real world, most patients with AIH are indeed on treatment, as were the current study participants, and we believe that it is impossible as well as unnecessary to dissect the impact of AIH itself and treatment on HrQoL. We needed to evaluate the entire HrQoL of all patients with AIH as a patient-reported outcome.

## Conclusions

The present study demonstrated HrQoL impairment in patients with AIH. In addition to disease progression, comorbidities, and treatment with prednisolone, and dosage of prednisolone were found to be associated with HrQoL impairment in patients with AIH. The proper care of patients with AIH should consider HrQoL, and specifically the factors identified in this study.

## Supporting information

S1 Table Chronic Liver Disease Questionnaire and 36-item short form survey scores among patients with autoimmune hepatitis without cirrhosis, patients with chronic hepatitis C, and healthy subjects. (DOCX)

S1 File Basic dataset of present study. (XLSX)
