# Supplementary material for: Health-related quality of life in patients with autoimmune hepatitis: A questionnaire survey
Source: PLoS One. 2018 Oct 4;13(10):e0204772. doi: 10.1371/journal.pone.0204772 (PMC6171853; doi:10.1371/journal.pone.0204772)
Supplement: S1 Table — (DOCX) [file pone.0204772.s001.docx]

**S1 Table.**

|  | AIH  (n=237) | CHC  (n=88) | Healthy  (n=97) | *P*-value  (AIH vs. CHC) | *P*-value  (AIH vs. Healthy) |
| --- | --- | --- | --- | --- | --- |
| CLDQ |  |  |  |  |  |
| Overall | 5.6 (4.9-6.0) | 5.6 (5.0-5.8) | 6.2 (5.7-6.5) | 0.918 | < 0.001 |
| Abdominal | 6.0 (5.3-6.7) | 6.0 (5.6-6.7) | 6.3 (5.7-7.0) | 0.321 | 0.119 |
| Fatigue | 5.2 (4.4-5.8) | 5.3 (4.6-5.8) | 5.8 (5.4-6.2) | 0.647 | < 0.001 |
| Systemic | 5.6 (4.8-6.2) | 5.6 (5.0-6.0) | 6.2 (5.8-6.6) | 0.783 | < 0.001 |
| Activity | 5.8 (5.0-6.4) | 6.0 (5.5-6.3) | 6.3 (6.0-7.0) | 0.546 | < 0.001 |
| Emotions | 5.4 (4.4-6.0) | 5.5 (4.8-5.9) | 5.9 (5.3-6.3) | 0.909 | < 0.001 |
| Worry | 5.4 (4.4-6.0) | 5.6 (4.6-6.0) | 6.8 (6.3-7.0) | 0.926 | <0.001 |
| SF-36 |  |  |  |  |  |
| Physical functioning | 90 (70-95) | 90 (75-95) | 95(90-100) | 0.797 | < 0.001 |
| Role physical | 89.6 (62.5-100) | 100 (75-100) | 100 (87.5-100) | 0.099 | < 0.001 |
| Bodily pain | 84 (62-100) | 74 (61-100) | 84 (72-100) | 0.094 | 0.071 |
| General health | 52 (45-62) | 53.5 (45-62) | 72 (62-84.5) | 0.471 | < 0.001 |
| Vitality | 62.5 (50-75) | 62.5 (50-68.8) | 75 (62.5-81.3) | 0.885 | < 0.001 |
| Social functioning | 100 (68.8-100) | 100 (75-100) | 100 (87.5-100) | 0.132 | < 0.001 |
| Role emotion | 91.7 (66.7-100) | 100 (83.3-100) | 100 (91.7-100) | 0.021 | < 0.001 |
| Mental health | 70 (55-85) | 75 (60-85) | 80 (70-90) | 0.428 | < 0.001 |
| PCS | 49.4 (38.6-54.9) | 47.1 (38.3-53.2) | 54.2 (49.6-57.2) | 0.225 | < 0.001 |
| MCS | 52.2 (44.4-57.3) | 51.3 (46.4-54.8) | 55.0 (48.2-60.5) | 0.265 | 0.005 |
| RCS | 49.7 (38.9-55.9) | 53.9 (47.0-58.0) | 51.8 (47.2-55.5) | 0.004 | 0.066 |

The data are expressed as the median with the interquartile range.

CLDQ, Chronic Liver Disease Questionnaire; SF-36, 36-Item Short Form Survey; AIH, autoimmune hepatitis; CHC, chronic hepatitis C; PCS, physical component summary; MCS, mental component summary; RCS, role/social component summary
